# Artificial Intelligence Drives Advances in Multi-Omics Analysis and Precision Medicine for Sepsis

**DOI:** 10.3390/biomedicines14020261

**Published:** 2026-01-23

**Authors:** Youxie Shen, Peidong Zhang, Jialiu Luo, Shunyao Chen, Shuaipeng Gu, Zhiqiang Lin, Zhaohui Tang

**Affiliations:** Department of Trauma Surgery, Emergency Surgery & Surgical Critical, Tongji Trauma Center, Tongji Hospital, Tongji Medical College, Huazhong University of Science and Technology, Wuhan 430030, China; shenyouxie@hust.edu.cn (Y.S.);

**Keywords:** sepsis, artificial intelligence, machine learning, multi-omics, precision medicine

## Abstract

Sepsis is a life-threatening syndrome characterized by marked clinical heterogeneity and complex host–pathogen interactions. Although traditional mechanistic studies have identified key molecular pathways, they remain insufficient to capture the highly dynamic, multifactorial, and systems-level nature of this condition. The advent of high-throughput omics technologies—particularly integrative multi-omics approaches encompassing genomics, transcriptomics, proteomics, and metabolomics—has profoundly reshaped sepsis research by enabling comprehensive profiling of molecular perturbations across biological layers. However, the unprecedented scale, dimensionality, and heterogeneity of multi-omics datasets exceed the analytical capacity of conventional statistical methods, necessitating more advanced computational strategies to derive biologically meaningful and clinically actionable insights. In this context, artificial intelligence (AI) has emerged as a powerful paradigm for decoding the complexity of sepsis. By leveraging machine learning and deep learning algorithms, AI can efficiently process ultra-high-dimensional and heterogeneous multi-omics data, uncover latent molecular patterns, and integrate multilayered biological information into unified predictive frameworks. These capabilities have driven substantial advances in early sepsis detection, molecular subtyping, prognosis prediction, and therapeutic target identification, thereby narrowing the gap between molecular mechanisms and clinical application. As a result, the convergence of AI and multi-omics is redefining sepsis research, shifting the field from descriptive analyses toward predictive, mechanistic, and precision-oriented medicine. Despite these advances, the clinical translation of AI-driven multi-omics approaches in sepsis remains constrained by several challenges, including limited data availability, cohort heterogeneity, restricted interpretability and causal inference, high computational demands, difficulties in integrating static molecular profiles with dynamic clinical data, ethical and governance concerns, and limited generalizability across populations and platforms. Addressing these barriers will require the establishment of standardized, multicenter datasets, the development of explainable and robust AI frameworks, and sustained interdisciplinary collaboration between computational scientists and clinicians. Through these efforts, AI-enabled multi-omics research may progress toward reproducible, interpretable, and equitable clinical implementation. Ultimately, the synergy between artificial intelligence and multi-omics heralds a new era of intelligent discovery and precision medicine in sepsis, with the potential to transform both research paradigms and bedside practice.

## 1. Introduction

**Figure 1:**
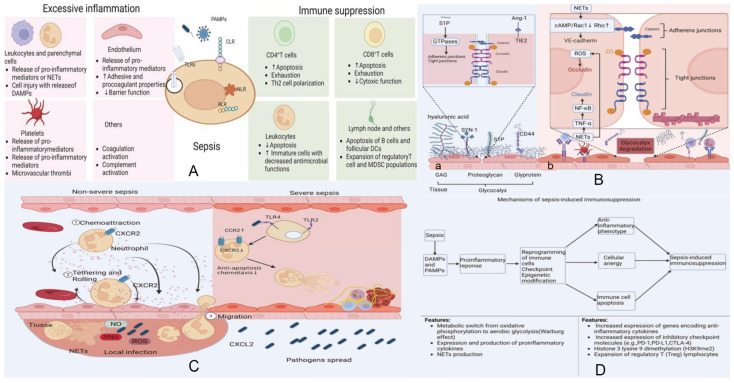
Integrated mechanisms underlying the host response and pathophysiology of sepsis. Data from [5]. (**A**) **Host immune activation and loss of homeostasis.** Upon pathogen invasion, innate immune cells together with epithelial and endothelial cells initiate early local defense through pattern-recognition receptors (PRRs), including TLRs, NLRs, RLRs, and CLRs. These pathways drive phagocyte recruitment, complement activation, and the release of inflammatory cytokines and chemokines, forming an effective protective immunity. In most infections, compensatory anti-inflammatory mechanisms restore immunological homeostasis. However, when pathogens overwhelm these responses, dysregulated inflammation and impaired resolution lead to tissue injury and the onset of sepsis. (**B**) **Endothelial injury and barrier dysfunction.** (**a**) Sepsis disrupts endothelial homeostasis primarily through degradation of the glycocalyx layer. NET-associated proteases and histone–DNA complexes, together with cytokines such as TNF-α, IL-6, and IL-8, accelerate glycocalyx breakdown. Loss of this protective barrier exposes adhesion molecules, enhances leukocyte and platelet attachment, and exacerbates inflammation. (**b**) Cleavage of junctional proteins—NF-κB-mediated claudin disruption, ROS-induced occludin redistribution, and protease-mediated VE-cadherin degradation—further increases vascular permeability and promotes endothelial apoptosis. (**C**) **Crosstalk between inflammation, coagulation, and complement.** Pathogens stimulate monocytes/macrophages to release cytokines and microvesicles, initiating both inflammatory and coagulation pathways. Neutrophils activated by cytokines release NETs, propagating endothelial damage and amplifying DAMP release. DAMP–PRR interactions reinforce cytokine production and trigger activation of coagulation factors VII and XII. Platelets sense DAMPs through TLRs, RAGE, and DC-SIGN, releasing inflammatory mediators such as C3. Complement activation culminates in membrane attack complex (MAC) formation, further injuring endothelial cells. Convergence of extrinsic and intrinsic coagulation pathways drives thrombin generation, which reinforces inflammation and results in inflammatory thrombosis. (**D**) **Mechanisms of sepsis-induced immunosuppression.** The host response in sepsis reflects a dynamic interplay between pro-inflammatory and anti-inflammatory programs, driven by PAMP- and DAMP-mediated immune activation. While excessive inflammation contributes to organ injury, simultaneous induction of anti-inflammatory pathways leads to immune cell exhaustion, apoptosis, epigenetic reprogramming, and functional paralysis. Persistence of these suppressive mechanisms results in prolonged immunosuppression, increasing susceptibility to secondary infections and impairing recovery. Adapted and re-organized from “Unveiling the research advances of sepsis: pathogenesis, precise intervention and clinical perspective” [5] under CC BY-SA 4.0 license.

Sepsis is a complex clinical syndrome defined by life-threatening organ dysfunction resulting from a dysregulated host response to infection [1]. It represents a substantial global healthcare burden, affecting millions of individuals each year and accounting for nearly one-fifth of all deaths worldwide [2,3]. At the molecular level, sepsis arises from highly interconnected disturbances involving immune dysregulation, coagulopathy, endothelial injury, metabolic reprogramming, and direct tissue damage [4,5,6,7,8] (Figure 1). Despite decades of intensive investigation, the precise pathophysiological underpinnings of sepsis and its underlying mechanisms remain incompletely elucidated.

Traditional mechanistic studies have substantially advanced the field by elucidating critical signaling pathways and identifying candidate biomarkers, including those related to Toll-like receptor signaling, nuclear factor-κB activation, and cytokine networks [6,7,8]. However, these approaches are typically hypothesis-driven and focus on isolated molecular pathways. While invaluable for dissecting specific mechanisms, such reductionist strategies are inherently limited in their ability to capture the nonlinear, dynamic, and multifactorial interactions that characterize sepsis pathophysiology.

In contrast, omics technologies enable comprehensive and unbiased profiling of molecular states across multiple biological layers, including the genome, transcriptome, proteome, and metabolome [9]. The integration of these modalities has facilitated a paradigm shift from single-molecule investigations to system-level analyses, allowing researchers to interrogate the coordinated interplay among genetic variation, gene expression, protein function, and metabolic activity [10]. These integrative approaches offer a more holistic view of sepsis biology and provide a foundation for molecular stratification, biomarker discovery, and precision medicine.

Nevertheless, the full potential of multi-omics approaches is constrained by substantial analytical and methodological challenges. Multi-omics datasets are typically high-dimensional, sparse, heterogeneous, and affected by technical and biological noise, rendering conventional statistical methods inadequate for effective integration and interpretation [10]. Artificial intelligence (AI), particularly machine learning and deep learning techniques, provides a flexible computational framework capable of modeling complex nonlinear relationships and extracting latent structure from large-scale datasets [11,12,13]. Importantly, the application of AI to multi-omics data should be viewed as a means to support hypothesis generation and biological discovery rather than as an inherently clinically deployable solution. Successful translation ultimately depends on rigorous validation, interpretability, and alignment with real-world clinical workflows.

In this review, we provide a comprehensive synthesis of omics-driven insights into sepsis pathophysiology, with a particular emphasis on the role of artificial intelligence in enabling integrative multi-omics analysis. We summarize representative applications in diagnosis, patient stratification, prognosis prediction, and therapeutic target discovery, as well as critically discuss current limitations and future directions for AI-driven precision medicine in sepsis.

## 2. Literature Search Strategy

The literature included in this review was identified through a structured search of major biomedical and interdisciplinary databases, primarily PubMed and Scopus. Searches were conducted to capture peer-reviewed articles published up to 2025.

The search strategy combined keywords related to sepsis, artificial intelligence, and omics technologies, including but not limited to the following: “sepsis,” “artificial intelligence,” “machine learning,” “deep learning,” “multi-omics,” “genomics,” “transcriptomics,” “proteomics,” “metabolomics,” “single cell,” and “precision medicine.” These terms were used alone or in combination with Boolean operators (AND/OR).

Additional relevant studies were identified by screening the reference lists of selected articles and recent reviews. Priority was given to original research articles, high-quality reviews, and consensus or guideline papers that addressed AI-driven omics analysis, methodological advances, or translational challenges in sepsis. Given the narrative nature of this review, formal inclusion or exclusion criteria and quantitative quality scoring were not applied.

## 3. Omics-Driven Insights into the Pathophysiology of Sepsis

The integration of multi-omics approaches has significantly advanced our understanding of sepsis by uncovering novel molecular mechanisms and identifying potential biomarkers [14,15,16]. In the subsequent discussion, we delineate the distinct contributions of genomics, transcriptomics, proteomics, metabolomics and single-cell omics to the advancement of our understanding of the pathophysiology and clinical management of sepsis (Table 1).

### 3.1. Genomics

Genomics deciphers DNA sequences to elucidate disease-linked genomic alterations, providing the foundation for precision diagnostics and therapeutics [17].

Two major technologies for genomic studies are whole-genome sequencing (WGS) and whole-exome sequencing (WES). Whole-genome sequencing (WGS) is particularly suitable for comprehensive analyses of complex diseases [18]. For instance, metagenomic next-generation sequencing (mNGS) based on WGS technology has demonstrated a high positivity rate of 88% when used to detect pathogens in blood or pus samples from patients with sepsis, which is significantly higher than that of traditional culture methods (26%) [19]. In contrast, whole-exome sequencing (WES) is well-suited for screening known pathogenic mutations. For example, in patients with burns complicated by sepsis, WES has identified mutations in the ENTPD1 gene (which is associated with purine metabolism), suggesting that metabolic abnormalities may increase the risk of infection [20]. These findings underscore the utility of genomic approaches in identifying infection sources and host susceptibility factors, laying the groundwork for mechanism-based stratification.

### 3.2. Transcriptomics

Transcriptomics, which implies the analysis of all the RNA transcripts present in a cell, has developed into an important vehicle for the comprehension of the intricate mechanisms underlying sepsis. Various studies have identified sepsis-related biomarkers, hub genes, and pathways through transcriptomic analysis, contributing to the understanding and treatment of sepsis [21,22].

Early transcriptomic studies were largely based on microarray technology, which enables the detection of pre-defined gene sequences. For example, Fu et al. used microarray technology to analyze three datasets (GSE28750, GSE64457, and GSE95233), suggesting that apoptosis of immune cells and imbalanced inflammation are key mechanisms in sepsis [23]. However, microarray technology can only detect known sequences. The emergence of RNA sequencing (RNA-seq) has overcome this limitation, revolutionizing transcriptomics by enabling unbiased capture and quantification of the entire transcriptome [24]. This method is particularly powerful for studying gene expression dynamics, revealing details about an organism’s biology, and identifying functions of previously unannotated genes [25]. Within RNA-seq, bulk transcriptomics has uncovered population-level dysregulation of immune response pathways, whereas single-cell RNA sequencing (scRNA-seq) has further resolved cellular heterogeneity and dysfunction with high resolution [26]. For instance, Ma et al. employed RNA-Seq technology to identify that the combination of miR-4772-5p-iso and miR-150 can be utilized to construct a “sepsis score,” which demonstrates a diagnostic accuracy rate of 86% [27]. Complementarily, Yao et al. utilized single-cell omics to identify a distinct monocyte subtype characterized by low HLA-DR expression and high S100A expression, which is associated with an immunosuppressive phenotype during sepsis [28]. Together, these findings illustrate the complementary strengths of bulk and single-cell transcriptomics: bulk approaches highlight overarching immune alterations, while single-cell analyses capture cell-specific mechanisms critical to sepsis pathophysiology.

### 3.3. Proteomics

As the cellular “workhorses,” proteins execute nearly all biological operations—DNA replication, signal transduction, metabolic catalysis, and motility—thereby forming the pivotal molecular nexus between genotype and phenotype and offering an authentic readout of cellular state [29]. The cornerstone analytical methodology in proteomics is mass spectrometry (MS) [30].

The application of proteomics in the fields of diagnosis, biomarker discovery, and treatment represents a significant advancement in medical science, offering promising avenues for improving disease management and patient outcomes. The human plasma proteome, for instance, contains a vast array of proteins that can serve as potential biomarkers for various diseases [31]. Tong et al. conducted a proteomic analysis of peripheral blood samples from 27 patients with sepsis, 11 with septic shock, and 24 non-sepsis ICU controls. Their findings demonstrated that high-mobility group box 1 (HMGB1), matrix metalloproteinase 8 (MMP8), neutrophil gelatinase-associated lipocalin (NGAL), lactotransferrin (LTF), and grancalcin (GCA) exhibit diagnostic utility for sepsis, with an area under the curve (AUC) exceeding 0.82 [32]. In addition to plasma proteins, urinary proteins represent a valuable diagnostic target. Su et al. demonstrated through proteomic profiling of urine samples from sepsis patients that low expression of lysosome-associated membrane protein-1 (LAMP-1) is predictive of increased 28-day mortality, with a specificity of 92% [33]. Proteomics also facilitates the identification of novel therapeutic targets for drug development [34]. For instance, targeting ferroptosis-related proteins, such as glutathione peroxidase 4 (GPX4) and acyl-CoA synthetase long-chain family member 4 (ACSL4), has been shown to mitigate organ injury and improve clinical outcomes in sepsis [35]. These observations support the utility of proteomic profiling in identifying accessible biomarkers and exploring pathophysiological mechanisms.

### 3.4. Metabolomics

Metabolomics is a high-throughput, comprehensive quantitative analysis of all endogenous and exogenous low-molecular-weight metabolites in cells, tissues, or biofluids [36]. Sepsis is frequently associated with a spectrum of metabolic disturbances. The detection and analysis of metabolites can provide a more comprehensive and nuanced understanding of the pathophysiology underlying sepsis [37].

Metabolomics employs various analytical techniques to study the complete set of metabolites in biological samples, with Mass Spectrometry (MS) and Nuclear Magnetic Resonance (NMR) spectroscopy being two prominent methods. MS is highly sensitive [38], making it suitable for targeted metabolomics studies and pathway activity prediction using algorithms like mummichog [39]. NMR is non-destructive, requires minimal sample preparation, and offers high reproducibility, making it effective for unambiguous identification of unknown metabolites [38].

In the context of disease diagnosis, metabolomics has shown promise in identifying novel biomarkers for early detection and monitoring of various conditions. Su et al. conducted a metabolomic analysis of serum samples from a cohort comprising 65 individuals, including 35 patients with sepsis, 15 patients with Systemic Inflammatory Response Syndrome (SIRS), and 15 healthy controls. Their study identified seven distinct metabolites that exhibited significant differences between the sepsis and control groups, thereby demonstrating potential utility as biomarkers for the diagnosis of sepsis [40]. The application of metabolomics extends beyond diagnosis to include therapeutic evaluation and management. By providing a comprehensive view of the metabolic state, metabolomics can aid in understanding the mechanisms of drug action and predicting individual variations in drug response phenotypes [41].

In recent years, the advent and continual refinement of omics technologies have substantially advanced the study of sepsis. By enabling integrative multi-omics analyses, researchers can achieve a systems-level understanding of the complex physiological and pathophysiological cascades that drive the syndrome, thereby overcoming the reductionist limitations of single-omic approaches. For example, Andre Mu and colleagues systematically integrated genomic, transcriptomic, proteomic, and metabolomic datasets to delineate both conserved and pathogen-specific responses of sepsis-causing bacteria [42]. However, multi-omics datasets are typically high-dimensional, sparse, heterogeneous, and often contain missing values, which complicates direct interpretation and integration [43]. Extracting meaningful features and formulating biologically plausible hypotheses from such complex datasets therefore remains a major challenge.

## 4. AI-Driven Approaches in Single-Omics and Multi-Omics Analysis

**Figure 2:**
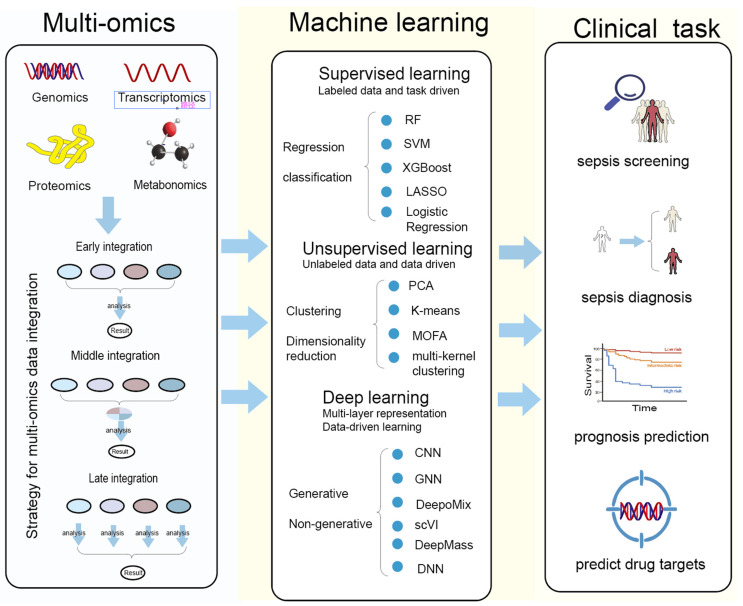
Multi-omics integration and machine-learning strategies for clinical applications in sepsis.

Artificial intelligence has emerged as a transformative tool in biomedical research by enabling the integration and interpretation of high-dimensional, heterogeneous datasets, thereby supporting data-driven discovery and translational research in complex diseases [44] (Figure 2).

This figure summarizes the workflow linking multi-omics data, machine-learning methodologies, and major clinical tasks in sepsis research. Multi-omics modalities—including genomics, transcriptomics, proteomics, and metabolomics—can be integrated through early, middle, or late fusion strategies to generate comprehensive biological representations. These datasets are subsequently analyzed using supervised learning (e.g., random forest, SVM, XGBoost, LASSO, logistic regression) for regression or classification tasks; unsupervised learning (e.g., PCA, K-means, MOFA, multi-kernel clustering) for clustering and dimensionality reduction; and deep-learning approaches (e.g., CNN, GNN, DeepoMix, scV1, DeepMass, DNN) for high-dimensional feature extraction and generative or non-generative modeling. The analytical outputs support key clinical applications, including sepsis screening, sepsis diagnosis, prognosis prediction, and the identification of potential therapeutic drug targets.

### 4.1. Artificial Intelligence for Single-Omics Data Analysis

AI has played a transformative role in genomic research. DeepVariant achieves high-precision identification of genetic variants by leveraging deep neural networks to model subtle sequence-context features, thereby substantially reducing both false-positive and false-negative mutation calls relative to conventional statistical approaches [45]. In parallel, the proposed HAICoGA (Human–AI Collaborative Genome Annotation) framework highlights the potential of combining AI systems with expert knowledge to enhance both the efficiency and accuracy of genome annotation [46].

Traditional bulk RNA-seq lacks spatial information, while current ST-seq methods have limitations in resolution and sensitivity. Tangram, a deep learning tool, integrates single-cell and single-nucleus RNA-seq data with spatially resolved transcriptomics through adversarial training and deep neural networks. It enhances the resolution and accuracy of spatial transcriptomics, enriches the spatial context of scRNA-seq data, and shows potential in elucidating chromatin accessibility patterns [47].

Artificial intelligence (AI) has been effectively applied in both proteomics and metabolomics, significantly enhancing the capabilities of these fields. In proteomics, AI methods are particularly well-suited for mass spectrometry-based analyses, especially in the identification of unknown peptides, where they have demonstrated high accuracy [48]. For instance, tools such as DeepMass utilize deep learning to predict peptide fragmentation patterns, thereby improving the precision of protein identification. Additionally, AI has been employed to elucidate protein–nucleic acid and protein–protein interactions, which are crucial for understanding various biological processes.

In the realm of metabolomics, Artificial intelligence (AI) is employed to surmount the challenges associated with metabolite representation [49], thereby offering valuable insights that advance sepsis research. AI has strengthened the data processing and analysis capabilities, facilitating the interpretation of complex metabolic networks [50]. AI-driven models can streamline biomarker discovery by identifying subtle metabolic shifts associated with diseases. Furthermore, AI can construct metabolic models, which can provide insights into metabolic pathways and their regulation.

In summary, artificial intelligence has made substantial contributions to diverse omics research endeavors, thereby significantly enhancing our capacity to elucidate and understand complex biological mechanisms.

### 4.2. AI-Based Integration Analysis of Multi-Omics Data

The integration of multi-omics data provides a systems-level perspective on the biological complexity of sepsis, enabling the joint analysis of molecular processes that span genomic variation, transcriptional regulation, protein function, metabolic activity, and clinical phenotypes derived from electronic health records. Importantly, the goal of multi-omics integration is not algorithmic sophistication per se, but the extraction of biologically coherent and clinically interpretable representations that are appropriate for specific research or translational objectives. Given the wide variability in cohort size, data completeness, temporal resolution, and technical noise across omics modalities, the choice of integration strategy must be guided primarily by data characteristics and study intent rather than by model complexity alone [51] (Table 2).

Conceptually, multi-omics integration strategies are commonly categorized into early, intermediate, and late integration, each corresponding to distinct analytical assumptions and practical constraints [52,53,54,55]. Early integration concatenates features across modalities and is best suited to small-scale, relatively homogeneous datasets with limited missingness, where cross-omics correlations are expected to be strong [52,53]. Intermediate integration methods, which infer shared latent representations through approaches such as autoencoders, variational models, or multi-kernel learning, are more appropriate for high-dimensional and heterogeneous datasets but require careful regularization and substantial computational resources [52,54]. Late integration, by contrast, adopts a modular framework in which modality-specific models are trained independently and combined at the decision level, offering greater robustness to missing data and modality imbalance at the expense of explicit feature-level interaction modeling [52,55]. In practice, late or hybrid integration strategies are often more feasible for sepsis research, particularly when combining molecular omics with real-world clinical data.

A growing number of AI-driven frameworks exemplify these integration strategies, often optimized for specific dataset types and scales. MOFA+ leverages unsupervised factor analysis to extract latent representations across omics layers, and is particularly effective in moderate-size datasets with multiple continuous modalities [56]. scVI and totalVI apply probabilistic deep learning to integrate transcriptomic and proteomic signals at the single-cell level, addressing the high sparsity and dropout rates inherent in these datasets [57,58]. DeepOmix employ autoencoder-based architectures for predictive modeling across genomics, transcriptomics, and metabolomics, handling datasets that combine wide feature spaces with relatively small sample sizes [59]. Multimodal attention networks such as MOMA enable adaptive weighting of heterogeneous datasets, which is critical when integrating large-scale clinical records with molecular omics [60]. Collectively, these AI tools have proven capable of addressing the intrinsic challenges of high dimensionality, sparsity, missingness, and heterogeneity that characterize sepsis datasets.

AI-based multi-omics integration not only resolves technical obstacles but also generates biologically meaningful and clinically actionable embeddings. By explicitly accounting for the types, sizes, and characteristics of different datasets, and transforming heterogeneous omics signals into coherent representations, these approaches lay the foundation for precision applications in sepsis. Such applications include early detection of infection onset, stratification of patients into molecularly defined endotypes, prediction of prognosis and therapeutic response, and discovery of novel treatment targets.

## 5. Application of AI in Sepsis Omics

The embedding derived from AI-based integration of omics data directly enable clinically actionable tasks. Here, we map these computational advances to four critical use cases: early detection, subtype stratification, prognosis and treatment response, and therapeutic target discovery (Table 3).

### 5.1. Early Sepsis Detection and Screening

Early identification of sepsis is a critical clinical priority, as delays in recognition and treatment are strongly associated with increased mortality [77,78]. In current clinical practice, AI-based prediction models derived from electronic health records (EHRs) represent the most feasible and scalable approach for real-time sepsis screening and early warning, owing to their reliance on routinely collected physiological and laboratory data. In this context, multi-omics-based AI models should not be viewed as direct competitors to EHR-based systems for frontline screening. Instead, their added value lies in providing complementary molecular information that may refine risk stratification, elucidate underlying biological mechanisms, or identify patient subgroups with distinct immunological or metabolic profiles.

Accordingly, the majority of existing sepsis prediction models have been constructed using clinical data, particularly EHRs. For instance, the InSight model employs a machine learning classification system to predict the onset of sepsis by leveraging a minimal set of variables extracted from EHR data. This model achieves performance that is competitive with existing scoring systems, such as the Quick Sequential Organ Failure Assessment (qSOFA) and the modified Early Warning Score (mEWS) [61]. Rosnati et al. propose MGP-AttTCN: a joint multitask Gaussian Process and attention-based deep learning model to early predict the occurrence of sepsis in an interpretable manner [62]. Kim et al. similarly developed an early prediction system, DeepSEPS, which is based on EHRs. This system has demonstrated superior performance compared to traditional scoring systems; the AUROC was 0.934 (at the time of sepsis) and the AUROC predicted 3 h in advance was 0.85 [63]. Sudarsan et al. also conducted a comparable study, utilizing on-chip artificial intelligence (AI) to amalgamate data from EMRs with physiological sensor data, specifically electrocardiogram (ECG) data. This fusion model achieves a late fusion accuracy of 92.2% in predicting sepsis onset 4 h before it occurs [64]. In parallel, predictive models based on omics data have demonstrated promising performance, albeit in more specialized research settings. For instance, Yagin et al. employed the KTBoost model in conjunction with SHAP (SHapley Additive exPlanations) analysis to identify that alterations in the concentrations of platelet metabolites, such as carnitine and glutamine, can serve as early warning indicators for sepsis, with the potential to predict its onset up to 6 h in advance. This approach achieved an area under the receiver operating characteristic curve (AUC) of 0.94 [65].

Overall, these findings suggest that while EHR-based AI systems remain the most practical tools for rapid, population-level sepsis detection, AI-driven analyses of omics and multi-omics data provide a secondary, mechanistically informed layer of insight that is better suited to precision medicine and pathophysiological discovery than to universal bedside deployment [79] (Table 4).

### 5.2. Diagnosis, Classification and Grading of Sepsis

Given the severe heterogeneity of sepsis, accurate diagnosis and precise identification of its subtypes are essential. AI-based sepsis omics can contribute to these goals. Moreover, AI-based sepsis omics is capable of elucidating molecular network abnormalities in sepsis and aiding in the identification of valid diagnostic markers. By investigating the disparities in gene expression between sepsis and normal populations, sepsis-related biomarkers can be identified. For instance, a recent study identified genes such as COMMD9, CSF3R, and NUB1 as potential biomarkers for predicting sepsis, which were subsequently confirmed through rigorous experimental validation, including quantitative real-time polymerase chain reaction (qRT-PCR) and Western Blot analysis [67]. Additionally, a four-gene signature, comprising the hub genes LCK, CCL5, ITGAM, and MMP9, has been established as a diagnostic model for sepsis [66]. One notable example of AI’s application in sepsis omics is the use of deep learning and transfer learning algorithms for analyzing two-dimensional polyacrylamide gel electrophoresis (HP-2D-PAGE) images of patient serum. This method achieved an accuracy of ≥98% in distinguishing sepsis patients from healthy individuals [68].

While the aforementioned biomarkers can aid in determining whether a patient has sepsis, merely diagnosing the presence of sepsis is insufficient. The risk of mortality varies significantly among different subtypes of sepsis. Therefore, it is essential to identify the subtypes of sepsis that are at a high risk of death and to provide corresponding interventions. For instance, unsupervised analysis of transcriptomics data across multiple datasets has revealed three robust clusters associated with different clinical severities and mortality rates in bacterial sepsis [69]. Similar studies have employed K-means cluster analysis based on routinely available clinical data to rapidly identify two subtypes of sepsis that are associated with distinct clinical outcomes (OR = 2.214) [70]. These findings underscore the potential of AI-driven clustering techniques to uncover molecular heterogeneity in sepsis, offering valuable insights into personalized treatment strategies. By identifying distinct molecular subtypes, AI models can facilitate the development of tailored therapeutic approaches that address the specific pathophysiological mechanisms underlying each subtype, thereby enhancing treatment efficacy and patient outcomes.

Moreover, the development of interpretable AI models, such as those incorporating attention mechanisms or SHAP analysis, has further enhanced the utility of these models in clinical decision-making. These models not only provide accurate predictions but also offer transparency into the decision-making process, allowing clinicians to better understand the underlying biological mechanisms and make more informed treatment decisions.

### 5.3. Prediction of Prognosis and Treatment Response in Sepsis

AI-based prediction models, constructed using omics or multimodal datasets, have demonstrated potential for early estimation of prognosis and treatment response in patients with sepsis. For example, random forest (RF) models have identified novel biomarker panels that outperform traditional markers such as procalcitonin (PCT) and C-reactive protein (CRP), facilitating early diagnosis and demonstrating higher predictive power for 30-day mortality risk [71]. Similarly, Hu et al. reported that an XGBoost model achieved an AUC of 0.884 and an accuracy of 89.5% in the validation cohort, highlighting the potential of machine learning algorithms to enhance the accuracy and efficiency of sepsis diagnosis and prognosis [72]. AI models have also been applied to metabolomics data, revealing metabolic pathways associated with patient survival and providing insights into the underlying pathophysiology [73].

Beyond prognostic assessment, AI approaches can support the optimization of treatment strategies. Models have been developed to improve pathogen identification for antibiotic selection [74,80], optimize dosing regimens through pharmacokinetic modeling [81], and predict antibiotic resistance to guide empirical therapy [82,83]. Furthermore, AI has been applied to critical management decisions such as fluid resuscitation, with models evaluating the impact of 24 h fluid volumes on mortality and informing individualized strategies [75,84].

### 5.4. Prediction of Drug Targets

AI-powered multi-omics integration serves as a robust platform for the systematic identification of novel therapeutic targets in sepsis. By leveraging high-dimensional omics data, artificial intelligence algorithms can extract salient features, reconstruct molecular interaction networks, and pinpoint driver genes, proteins, or metabolic pathways that are critically involved in disease progression.

For example, Huang et al. implemented an AI-driven multi-omics integration approach to investigate sepsis development in patients with severe burns. Their study employed computational models to integrate genomic, transcriptomic, and proteomic data, successfully identifying seven susceptibility genes, four microRNAs, and two proteins as potential early biomarkers for burn-induced sepsis. Notably, the protein S100A8 was experimentally validated to function both as a diagnostic indicator and a prospective therapeutic target [20].

Beyond target identification, AI-enabled integrative analyses can support the interpretation of candidate targets within the broader metabolic and molecular landscape of sepsis. Metabolomic profiling studies have shown that profound metabolic reprogramming is associated with disease severity and clinical outcomes, suggesting that these alterations capture key features of sepsis pathophysiology [76]. Integrating such metabolomic information with other omics layers enables candidate targets to be viewed within coordinated disease-associated networks, thereby informing pathway-oriented therapeutic strategies. In this regard, AI-driven host-response profiling is best viewed as complementary to rapid molecular diagnostics, providing biological context that may inform treatment prioritization and stewardship rather than real-time pathogen detection.

At higher resolution, the integration of artificial intelligence with single-cell omics and microfluidic technologies is advancing target discovery to the level of cellular subpopulations. Through the analysis of single-cell gene expression profiles, AI algorithms can detect aberrantly expressed genes within specific immune cell subsets, enabling the identification of more precise and selective therapeutic targets with reduced risk of systemic adverse effects [85]. For instance, membrane proteins or signaling molecules that are highly expressed in distinct immune cell populations represent promising candidates for the development of novel monoclonal antibodies or small-molecule inhibitors.

## 6. Limitation and Future Directions

Despite the rapid methodological advances and growing body of literature, the clinical translation of AI-driven multi-omics approaches in sepsis remains confronted with substantial conceptual, technical, and practical challenges [86]. Addressing these limitations is essential to ensure that future developments move beyond proof-of-concept studies toward robust, reproducible, and clinically meaningful applications (Table 5).

### 6.1. Data Dependency and Methodological Robustness

Current AI models, particularly deep learning approaches, often require large volumes of high-quality labeled data to achieve robust performance [87]. In sepsis research, however, such datasets remain limited, and evolving diagnostic definitions (e.g., Sepsis-2 [88] to Sepsis-3 [1]) introduce additional heterogeneity in labeling across studies. Additionally, manual annotation of clinical and molecular data is time-consuming and requires expert knowledge, limiting dataset scalability. This issue is even more pronounced in multi-omics studies with small cohort sizes and high-dimensional molecular features, increasing the risk of overfitting, especially when feature selection and model training are performed on the same dataset.

AI-driven multi-omics models are often developed using retrospective, single-center cohorts and evaluated with AUROC metrics, which may overestimate performance and limit generalizability. Future studies should focus on few-shot and self-supervised learning techniques to reduce reliance on large labeled datasets [89]. Furthermore, rigorous validation strategies-including nested cross-validation, separation of feature selection from model training, and evaluation across independent cohorts-are crucial. Currently, AI-driven multi-omics models in sepsis should be seen as hypothesis-generating tools, not clinically actionable decision-support systems.

### 6.2. Interpretability, Causality, and Biological Relevance

Limited explainability remains a major barrier to clinical adoption [90]. Most models function as “black boxes,” making it difficult for clinicians to understand the basis of predictions. Expectations for interpretability vary across stakeholders: clinicians emphasize actionable insights, whereas developers focus on algorithmic transparency and predictive performance [91].

Explainable AI techniques, such as SHAP and attention mechanisms [92,93], can clarify the relative contribution of omics features and provide biological insight. However, these outputs primarily reflect statistical associations rather than causal relationships. In sepsis, where immune, metabolic, and endothelial pathways are highly interconnected and dynamically regulated, equating feature importance with mechanistic relevance can be misleading. Interpretability should therefore be framed as a tool to prioritize candidate genes, proteins, or pathways for subsequent functional validation, longitudinal studies, or formal causal inference analyses.

### 6.3. Computational Demands and Integration of Static and Dynamic Data

Training large-scale AI models requires substantial computational resources, limiting feasibility for many institutions. Model compression techniques, such as pruning and knowledge distillation [94,95], alongside distributed training, can reduce computational costs and accelerate development. Nevertheless, most current frameworks remain resource-intensive and are optimized for research rather than routine clinical deployment.

Another major challenge is integrating static molecular measurements with dynamic clinical trajectories. Multi-omics profiles are often collected at discrete time points, whereas clinical variables evolve continuously. Temporal misalignment, differing noise structures, and resolution disparities complicate multimodal data fusion. Approaches such as temporal anchoring, aggregation of longitudinal EHR data, or modality-specific representation learning followed by late-stage fusion remain exploratory and lack standardized validation, representing a key methodological hurdle.

### 6.4. Ethical, Security, and Governance Considerations

AI deployment in healthcare poses ethical, legal, and security risks (privacy breaches, misuse, algorithmic bias), necessitating robust legal frameworks, ethical guidelines, and self-auditing systems to ensure responsible implementation [44,96]. Equity and accessibility must be prioritized to ensure AI precision tools benefit diverse patient populations. Mitigation strategies include transparent reporting of cohort characteristics, stratified performance evaluation across demographic and clinical subgroups, and the use of reweighting or fairness-aware learning techniques [97,98].

From a translational perspective, practical barriers (high cost, logistical burden of omics profiling, limited feasibility of advanced modalities) hinder near-term clinical adoption of AI-driven multi-omics approaches. Single-cell technologies, despite superior biological resolution, are particularly challenging for acute sepsis care due to requirements for specialized infrastructure, long processing time, and high costs [26].

For acute sepsis management, prospective testing of AI multi-omics models must not disrupt time-sensitive clinical decisions. Thus, evaluations should adopt observational/parallel-layer designs, with omics samples collected without altering standard care, and AI outputs generated offline or blinded. Such “silent mode” or shadow deployments enable real-time data acquisition and outcome linkage without impacting clinical management.

Finally, compliance with software-as-a-medical-device regulatory frameworks, emphasizing transparency, reproducibility, external validation, and post-deployment monitoring, is also critical.

### 6.5. Limited Generalizability

Many AI-driven multi-omics models show strong performance in controlled research settings but generalize poorly to real-world clinical environments. In sepsis research, this limitation is compounded by cohort heterogeneity and, critically, by batch effects and platform variability arising from differences in sample processing, sequencing or proteomics technologies, and data preprocessing pipelines [99,100].The stability of AI-defined subtypes across populations remains an open question, highlighting that models trained in one cohort may not reliably reproduce molecular subtypes or predictions in another.

Statistical harmonization methods, such as ComBat, can partially mitigate these effects but often fail to fully remove cross-study bias, particularly in multi-omics integration [101]. Emerging AI-based strategies, including domain adaptation, transfer learning, and representation learning, aim to extract platform-invariant features and improve model robustness. However, their effectiveness in sepsis-related multi-omics applications has yet to be systematically validated across diverse, multicenter cohorts. Consequently, rigorous external validation and standardized data-generation and preprocessing protocols remain essential for improving generalizability.

## 7. Conclusions

Sepsis remains one of the most challenging syndromes in critical care medicine, owing to its profound biological heterogeneity, dynamic clinical trajectories, and context-dependent host responses. The convergence of AI with multi-omics technologies has created unprecedented opportunities to interrogate this complexity across molecular layers, enabling the transformation of high-dimensional and inherently noisy datasets into biologically meaningful representations. Collectively, advances in AI-driven genomics, transcriptomics, proteomics, metabolomics, and integrative multi-omics analyses have substantially deepened mechanistic understanding of sepsis, facilitated molecular stratification beyond conventional clinical phenotypes, and supported biomarker discovery as well as precision-oriented hypothesis generation.

Importantly, the current value of AI-driven multi-omics in sepsis should be interpreted through a realistic translational lens. Most existing models remain exploratory, and their primary contribution lies not in immediate bedside decision support, but in redefining disease taxonomy, enriching clinical trial design, and elucidating coordinated immune–metabolic–endothelial networks that underlie divergent clinical trajectories. In this context, AI functions less as a replacement for clinical judgment and more as a knowledge-generating engine that augments human expertise by guiding mechanistic inquiry and precision hypothesis testing.

Future progress will depend on a paradigm shift from isolated model development toward integrated and collaborative research ecosystems. Methodological innovation must be accompanied by standardized data-generation frameworks, transparent and interpretable modeling strategies, and closer alignment with real-world clinical workflows. Equally essential is the incorporation of ethical governance and equity-oriented design principles to ensure that AI-enabled precision approaches are reproducible, trustworthy, and applicable across diverse healthcare settings, rather than confined to highly specialized research environments.

Ultimately, the successful translation of AI-driven multi-omics in sepsis will require sustained interdisciplinary collaboration among clinicians, computational scientists, biologists, and policymakers. By harmonizing technical rigor with biological insight, clinical relevance, and ethical oversight, the field can move beyond descriptive analyses toward a mature, systems-level understanding of sepsis. Such coordinated efforts hold the promise of transforming AI-driven multi-omics from an academic innovation into a practical foundation for precise, patient-centered sepsis care.

## Figures and Tables

**Table 1:** Representative Biomarkers Identified by AI-Driven Omics Studies in Sepsis.

Omics Layer	Biomarker	Biological Relevance	Reported Clinical Association
Transcriptomics	LCK	T-cell receptor signaling	Immune suppression, mortality
Transcriptomics	CCL5	Leukocyte trafficking	Sepsis diagnosis
Transcriptomics	CSF3R	Neutrophil activation	Disease severity
Proteomics	HMGB1	Inflammatory mediator	Organ failure, prognosis
Proteomics	NGAL	Kidney injury marker	AKI, mortality
Metabolomics	Carnitine	Mitochondrial metabolism	Early metabolic reprogramming
Metabolomics	Lactate-related pathways	Energy imbalance	Shock and mortality

**Table 2:** Recommended Multi-Omics Integration Strategies Based on Study Design.

Study Characteristic	Early Integration (Feature-Level)	Intermediate Integration (Representation-Level)	Late Integration (Decision-Level)
Cohort size	Small (<200)	Moderate (200–1000)	Small to large
Missing data tolerance	Low	Moderate	High
Platform heterogeneity	Low	Moderate	High
Primary objective	Mechanistic discovery	Subtyping, prediction	Clinical prediction
Overfitting risk	High	Moderate	Low

**Table 3:** Summary of the application of AI-based in sepsis precision medicine.

Application	Omics/Data Source	AI/Method Used	Key Findings/Performance	Reference
Early Detection & Screening	EHR + physiological data	InSight (ML classifier)	Competitive with qSOFA/mEWS	Desautels et al. [61]
	EHR	MGP-AttTCN	Interpretable early sepsis prediction	Rosnati et al. [62]
	EHR	DeepSEPS	AUROC = 0.934 (onset), 0.85 (3 h pre-onset)	Kim et al. [63]
	EMR + ECG	On-chip fusion model	92.2% accuracy (4 h pre-onset)	Sadasivuni et al. [64]
	Metabolomics (platelet)	KTBoost, SHAP	Carnitine/glutamine as early warning markers; AUC = 0.94 (6 h pre-onset)	Yagin et al. [65]
Diagnosis & Subtyping	Transcriptomics	SVM, WGCNA	Four-gene signature (LCK, CCL5, ITGAM, MMP9)	Li et al. [66]
	Transcriptomics	SVM-RFE, LASSO regression model	COMMD9, CSF3R, NUB1 as diagnostic biomarkers	Wang et al. [67]
	Proteomics (serum 2D-PAGE)	CNN, Transfer Learning	≥98% accuracy in sepsis vs. healthy	Hayashi et al. [68]
	Transcriptomics (multi-dataset)	Unsupervised clustering	Three clusters with distinct mortality risks	Sweeney et al. [69]
	Clinical data	K-means clustering	Two subtypes with OR = 2.214 for mortality	Hu et al. [70]
Prognosis & Treatment Response	Multi-omics + EHR	Random Forest	Outperformed PCT/CRP for 30-day mortality	Wu et al. [71]
	EHR	XGBoost, SHAP	AUC = 0.884, Accuracy = 89.5%	Hu et al. [72]
	Metabolomics	FDA, GBM, LR, NSC, PLS-DA, LDA	Thirteen sepsis survival-associated metabolites were identified, including four novel ones (3-hydroxyisobutyrate, indole-3-acetic acid, fucose, glycochenodeoxycholic acid sulfate).	Kosyakovsky et al. [73]
	Raman spectroscopy dataset	CNN	Accurately identify 30 common bacterial pathogens (≥82% isolate-level accuracy even with low signal-to-noise spectra).	Ho et al. [74]
	EHR	a dynamic Marginal Structural Model	Targeted fluid restriction (6–10 L total, 8 L threshold) likely decreases 30-day mortality relative to standard care.	Shahn et al. [75]
Drug Target Discovery	Multi-omics (genomics, proteomics, metabolomics)	PCA, Cluster analysis	7 genes, 4 miRNAs, 2 proteins as early warning markers in burn sepsis	Huang et al. [20]
	Proteomics + Metabolomics	Multivariate integration model + clustering analysis	Fatty acid β-oxidation pathway is a potential therapeutic target; metabolic/proteomic alterations guide pathway-oriented intervention	Langley, R.J., et al. [76]

**Table 4:** Comparison of EHR-Based AI Models and AI-Driven Multi-Omics Models in Sepsis.

	EHR-Based Models	AI-Driven Multi-Omics Models
Primary clinical role	Real-time screening and early warning	Molecular stratification and mechanism elucidation
Data source	Vital signs, laboratory tests, clinical notes	Genomics, transcriptomics, proteomics, metabolomics
Data availability	Widely available in routine care	Limited to research or specialized centers
Cost per patient	Low	High
Bedside feasibility	Immediate, real-time	Currently limited, offline analysis
Temporal resolution	High-frequency, longitudinal	Mostly static snapshots
Mechanistic insight	Low (phenotypic)	High (pathway- and network-level)
Utility for drug discovery	Minimal	Substantial
Intended clinical use	Universal screening	Targeted precision medicine and trial enrichment

**Table 5:** Advantages of Artificial Intelligence Compared with Conventional Approaches in Sepsis Research and Management.

Clinical/Research Aspect	Conventional Approaches	AI-Based Approaches	Added Value of AI in Sepsis
Data handling	Limited capacity for high-dimensional data processing; reliance on pre-defined variables	Analysis of high-dimensional, heterogeneous, multimodal datasets	Integrates complex multi-omics and clinical data beyond human analytical limits
Feature discovery	Hypothesis-driven; focused on known biomarkers or pathways	Data-driven discovery of latent patterns and novel features	Reveals previously unrecognized molecular signatures and disease endotypes
Molecular subtyping	Based on single biomarkers or clinical phenotypes	Unsupervised or semi-supervised clustering across multiple omics layers	Captures biological heterogeneity and enables molecularly informed patient stratification
Prognosis prediction	Rule-based scoring systems (e.g., SOFA, qSOFA) with limited personalization	Machine learning models capturing nonlinear relationships among variables	Improves risk stratification and individualized outcome prediction
Multi-omics integration	Manual or statistical integration with limited scalability	Automated early-, intermediate-, or late-fusion strategies	Enables systems-level interpretation of host immune–metabolic responses
Pathogen identification	Culture-based or targeted molecular diagnostics	AI-assisted analysis of mNGS, spectroscopy, or omics-derived data	Facilitates broad-spectrum detection and clarification of mixed infections and resistance patterns
Drug target discovery	Reductionist, pathway-by-pathway exploration	Network-based, multi-omics-driven target prioritization	Identifies upstream regulators within disease-associated molecular networks
Temporal pattern recognition	Static snapshots and threshold-based interpretation	Learning of complex temporal and nonlinear disease dynamics	Enhances understanding of disease progression and treatment-response trajectories
Scalability	Labor-intensive and highly expert-dependent	Scalable after model training	Efficiently supports large-cohort and multicenter analyses
Clinical feasibility	Widely deployable but biologically superficial	Currently largely confined to research settings	Particularly valuable for hypothesis generation, trial stratification, and precision medicine research

## Data Availability

No new data were created or analyzed in this study. Data sharing is not applicable to this article.

## Abbreviations

AI	artificial intelligence
Ang-1	angiopoietin-1
AUC	area under the curve
CLRs	C-type lectin receptors
CNN	convolutional neural network
CRP	C-reactive protein
DAMPs	damage-associated molecular patterns
DC-SIGN	dendritic cell-specific intercellular adhesion molecule-3-grabbing non-integrin
DNN	deep neural network
DNA	deoxyribonucleic acid
ECG	electrocardiogram
EHR	electronic health record
FVIIa	factor VIIa
GAG	glycosaminoglycan
GNN	graph neural network
HMGB1	high-mobility group box 1
ICU	intensive care unit
IL	interleukin
LAMP-1	lysosome-associated membrane protein 1
LASSO	least absolute shrinkage and selection operator
LPS	lipopolysaccharide
MAC	membrane attack complex
ML	machine learning
mNGS	metagenomic next-generation sequencing
MOFA	multi-omics factor analysis
MMP8	matrix metalloproteinase 8
MS	mass spectrometry
NETs	neutrophil extracellular traps
NF-κB	nuclear factor kappa B
NLRs	NOD-like receptors
NMR	nuclear magnetic resonance
PAMPs	pathogen-associated molecular patterns
PCA	principal component analysis
PCT	procalcitonin
PD-1	programmed cell death protein 1
PD-L1	programmed death-ligand 1
PRRs	pattern-recognition receptors
qRT-PCR	quantitative real-time polymerase chain reaction
qSOFA	quick sequential organ failure assessment
RAGE	receptor for advanced glycation end products
RF	random forest
RNA-seq	RNA sequencing
ROS	reactive oxygen species
scRNA-seq	single-cell RNA sequencing
SHAP	Shapley additive explanations
SIRS	systemic inflammatory response syndrome
SVM	support vector machine
TIE2	tyrosine kinase with immunoglobulin-like and EGF-like domains 2
TLRs	Toll-like receptors
VE-cadherin	vascular endothelial cadherin
WES	whole-exome sequencing
WGCNA	weighted gene co-expression network analysis
WGS	whole-genome sequencing
XGBoost	extreme gradient boosting
ZO	zonula occludens

## References

[1] Singer M., Deutschman C.S., Seymour C.W., Shankar-Hari M., Annane D., Bauer M., Bellomo R., Bernard G.R., Chiche J.D., Coopersmith C.M. (2016). The Third International Consensus Definitions for Sepsis and Septic Shock (Sepsis-3). JAMA.

[2] Fleischmann C., Scherag A., Adhikari N.K., Hartog C.S., Tsaganos T., Schlattmann P., Angus D.C., Reinhart K. (2016). Assessment of Global Incidence and Mortality of Hospital-treated Sepsis. Current Estimates and Limitations. Am. J. Respir. Crit. Care Med..

[3] Rudd K.E., Johnson S.C., Agesa K.M., Shackelford K.A., Tsoi D., Kievlan D.R., Colombara D.V., Ikuta K.S., Kissoon N., Finfer S. (2020). Global, regional, and national sepsis incidence and mortality, 1990–2017: Analysis for the Global Burden of Disease Study. Lancet.

[4] Jarczak D., Kluge S., Nierhaus A. (2021). Sepsis—Pathophysiology and Therapeutic Concepts. Front. Med..

[5] Cheng L., Cao Y., Liu S., Lv L., Zhang J., Bao J., Wang G., Xu P. (2025). Unveiling the research advances of sepsis: Pathogenesis, precise intervention and clinical perspective. Int. J. Surg..

[6] Chousterman B.G., Swirski F.K., Weber G.F. (2017). Cytokine storm and sepsis disease pathogenesis. Semin. Immunopathol..

[7] Cohen J. (2002). The immunopathogenesis of sepsis. Nature.

[8] van der Poll T., van de Veerdonk F.L., Scicluna B.P., Netea M.G. (2017). The immunopathology of sepsis and potential therapeutic targets. Nat. Rev. Immunol..

[9] Qiao J., Cui L. (2022). Multi-Omics Techniques Make it Possible to Analyze Sepsis-Associated Acute Kidney Injury Comprehensively. Front. Immunol..

[10] Hasin Y., Seldin M., Lusis A. (2017). Multi-omics approaches to disease. Genome Biol..

[11] Agarwal N., Nupur, Paul P.K., Mishra S.K., Mani I., Singh V. (2024). Artificial Intelligence and Machine Learning for Analysis of Multi-omics. Multi-Omics Analysis of the Human Microbiome: From Technology to Clinical Applications.

[12] Averbuch T., Sullivan K., Sauer A., Mamas M.A., Voors A.A., Gale C.P., Metra M., Ravindra N., Van Spall H.G.C. (2022). Applications of artificial intelligence and machine learning in heart failure. Eur. Heart J. Digit. Health.

[13] Gao Y., Chen H., Wu R., Zhou Z. (2025). AI-driven multi-omics profiling of sepsis immunity in the digestive system. Front. Immunol..

[14] Scicluna B.P., van Vught L.A., Zwinderman A.H., Wiewel M.A., Davenport E.E., Burnham K.L., Nürnberg P., Schultz M.J., Horn J., Cremer O.L. (2017). Classification of patients with sepsis according to blood genomic endotype: A prospective cohort study. Lancet Respir. Med..

[15] Seymour C.W., Kennedy J.N., Wang S., Chang C.H., Elliott C.F., Xu Z., Berry S., Clermont G., Cooper G., Gomez H. (2019). Derivation, Validation, and Potential Treatment Implications of Novel Clinical Phenotypes for Sepsis. JAMA.

[16] Davenport E.E., Burnham K.L., Radhakrishnan J., Humburg P., Hutton P., Mills T.C., Rautanen A., Gordon A.C., Garrard C., Hill A.V. (2016). Genomic landscape of the individual host response and outcomes in sepsis: A prospective cohort study. Lancet Respir. Med..

[17] Riess O., Sturm M., Menden B., Liebmann A., Demidov G., Witt D., Casadei N., Admard J., Schütz L., Ossowski S. (2024). Genomes in clinical care. npj Genom. Med..

[18] Brittain H.K., Scott R., Thomas E. (2017). The rise of the genome and personalised medicine. Clin. Med..

[19] Pan T.J., Luo W.W., Zhang S.S., Xie J.Y., Xu Z., Zhong Y.Y., Zou X.F., Gong H.J., Ye M.L. (2024). The clinical application value of multi-site mNGS detection of patients with sepsis in intensive care units. BMC Infect. Dis..

[20] Huang J., Chen Y., Guo Z., Yu Y., Zhang Y., Li P., Shi L., Lv G., Sun B. (2023). Prospective study and validation of early warning marker discovery based on integrating multi-omics analysis in severe burn patients with sepsis. Burn. Trauma.

[21] Wong H.R., Marshall J.C. (2017). Leveraging Transcriptomics to Disentangle Sepsis Heterogeneity. Am. J. Respir. Crit. Care Med..

[22] Wu Y., Zhang L., Zhang Y., Zhen Y., Liu S. (2018). Bioinformatics analysis to screen for critical genes between survived and non-survived patients with sepsis. Mol. Med. Rep..

[23] Fu Q., Yu W., Fu S., Chen E., Zhang S., Liang T.B. (2020). Screening and identification of key gene in sepsis development: Evidence from bioinformatics analysis. Medicine.

[24] Lowe R., Shirley N., Bleackley M., Dolan S., Shafee T. (2017). Transcriptomics technologies. PLoS Comput. Biol..

[25] Hrdlickova R., Toloue M., Tian B. (2017). RNA-Seq methods for transcriptome analysis. Wiley Interdiscip. Rev. RNA.

[26] Wu X., Yang X., Dai Y., Zhao Z., Zhu J., Guo H., Yang R. (2024). Single-cell sequencing to multi-omics: Technologies and applications. Biomark. Res..

[27] Ma Y., Vilanova D., Atalar K., Delfour O., Edgeworth J., Ostermann M., Hernandez-Fuentes M., Razafimahatratra S., Michot B., Persing D.H. (2013). Genome-wide sequencing of cellular microRNAs identifies a combinatorial expression signature diagnostic of sepsis. PLoS ONE.

[28] Yao R.Q., Zhao P.Y., Li Z.X., Liu Y.Y., Zheng L.Y., Duan Y., Wang L., Yang R.L., Kang H.J., Hao J.W. (2023). Single-cell transcriptome profiling of sepsis identifies HLA-DR(low)S100A(high) monocytes with immunosuppressive function. Mil. Med. Res..

[29] Diz A.P., Martínez-Fernández M., Rolán-Alvarez E. (2012). Proteomics in evolutionary ecology: Linking the genotype with the phenotype. Mol. Ecol..

[30] Pukala T.L., Chen H. (2021). Editorial: Technical and Methodological Advances in Proteomics. Front. Chem..

[31] Anderson N.L., Anderson N.G. (2002). The Human Plasma Proteome: History, Character, and Diagnostic Prospects. Mol. Cell. Proteom..

[32] Tong Y., Ku X., Wu C., Liu J., Yang C., Tang W., Yan W., Tang J. (2019). Data-independent acquisition-based quantitative proteomic analysis reveals differences in host immune response of peripheral blood mononuclear cells to sepsis. Scand. J. Immunol..

[33] Su L., Cao L., Zhou R., Jiang Z., Xiao K., Kong W., Wang H., Deng J., Wen B., Tan F. (2013). Identification of novel biomarkers for sepsis prognosis via urinary proteomic analysis using iTRAQ labeling and 2D-LC-MS/MS. PLoS ONE.

[34] Moseley F.L., Bicknell K.A., Marber M.S., Brooks G. (2007). The use of proteomics to identify novel therapeutic targets for the treatment of disease. J. Pharm. Pharmacol..

[35] Zeng Z., Deng J., Wang G., Luo Z., Xiao W., Xie W., Liu J., Li K. (2025). Ferroptosis-related protein biomarkers for diagnosis, differential diagnosis, and short-term mortality in patients with sepsis in the intensive care unit. Front. Immunol..

[36] Sinem N., Sinem N., Hakima A. (2019). Metabolomics: Basic Principles and Strategies. Molecular Medicine.

[37] Pandey S. (2024). Sepsis, Management & Advances in Metabolomics. Nanotheranostics.

[38] Emwas A.H., Roy R., McKay R.T., Tenori L., Saccenti E., Gowda G.A.N., Raftery D., Alahmari F., Jaremko L., Jaremko M. (2019). NMR Spectroscopy for Metabolomics Research. Metabolites.

[39] Chong J., Soufan O., Li C., Caraus I., Li S., Bourque G., Wishart D.S., Xia J. (2018). MetaboAnalyst 4.0: Towards more transparent and integrative metabolomics analysis. Nucleic Acids Res..

[40] Su L., Huang Y., Zhu Y., Xia L., Wang R., Xiao K., Wang H., Yan P., Wen B., Cao L. (2014). Discrimination of sepsis stage metabolic profiles with an LC/MS-MS-based metabolomics approach. BMJ Open Respir. Res..

[41] Kaddurah-Daouk R., Kristal B.S., Weinshilboum R.M. (2008). Metabolomics: A global biochemical approach to drug response and disease. Annu. Rev. Pharmacol. Toxicol..

[42] Mu A., Klare W.P., Baines S.L., Ignatius Pang C.N., Guérillot R., Harbison-Price N., Keller N., Wilksch J., Nhu N.T.K., Phan M.-D. (2023). Integrative omics identifies conserved and pathogen-specific responses of sepsis-causing bacteria. Nat. Commun..

[43] Morabito A., De Simone G., Pastorelli R., Brunelli L., Ferrario M. (2025). Algorithms and tools for data-driven omics integration to achieve multilayer biological insights: A narrative review. J. Transl. Med..

[44] Topol E.J. (2019). High-performance medicine: The convergence of human and artificial intelligence. Nat. Med..

[45] Poplin R., Chang P.C., Alexander D., Schwartz S., Colthurst T., Ku A., Newburger D., Dijamco J., Nguyen N., Afshar P.T. (2018). A universal SNP and small-indel variant caller using deep neural networks. Nat. Biotechnol..

[46] Li X., Whan A., McNeil M., Starns D., Irons J., Andrew S.C., Suchecki R. (2025). A conceptual framework for human-AI collaborative genome annotation. Brief Bioinform..

[47] Biancalani T., Scalia G., Buffoni L., Avasthi R., Lu Z., Sanger A., Tokcan N., Vanderburg C.R., Segerstolpe Å., Zhang M. (2021). Deep learning and alignment of spatially resolved single-cell transcriptomes with Tangram. Nature Methods.

[48] Frejno M., Berger M.T., Tüshaus J., Hogrebe A., Seefried F., Graber M., Samaras P., Ben Fredj S., Sukumar V., Eljagh L. (2025). Unifying the analysis of bottom-up proteomics data with CHIMERYS. Nat. Methods.

[49] Giera M., Yanes O., Siuzdak G. (2022). Metabolite discovery: Biochemistry’s scientific driver. Cell Metab..

[50] Xu Y., Jiang X., Hu Z. (2025). Synergizing metabolomics and artificial intelligence for advancing precision oncology. Trends Mol. Med..

[51] Martínez-García M., Hernández-Lemus E. (2022). Data Integration Challenges for Machine Learning in Precision Medicine. Front. Med..

[52] Rappoport N., Shamir R. (2019). Multi-omic and multi-view clustering algorithms: Review and cancer benchmark. Nucleic Acids Res..

[53] Picard M., Scott-Boyer M.P., Bodein A., Périn O., Droit A. (2021). Integration strategies of multi-omics data for machine learning analysis. Comput. Struct. Biotechnol. J..

[54] Huang S., Chaudhary K., Garmire L.X. (2017). More Is Better: Recent Progress in Multi-Omics Data Integration Methods. Front. Genet..

[55] Wu C., Zhou F., Ren J., Li X., Jiang Y., Ma S. (2019). A Selective Review of Multi-Level Omics Data Integration Using Variable Selection. High-Throughput.

[56] Argelaguet R., Arnol D., Bredikhin D., Deloro Y., Velten B., Marioni J.C., Stegle O. (2020). MOFA+: A statistical framework for comprehensive integration of multi-modal single-cell data. Genome Biol..

[57] Lopez R., Regier J., Cole M.B., Jordan M.I., Yosef N. (2018). Deep generative modeling for single-cell transcriptomics. Nat. Methods.

[58] Stuart T., Butler A., Hoffman P., Hafemeister C., Papalexi E., Mauck W.M., Hao Y., Stoeckius M., Smibert P., Satija R. (2019). Comprehensive Integration of Single-Cell Data. Cell.

[59] Zhao L., Dong Q., Luo C., Wu Y., Bu D., Qi X., Luo Y., Zhao Y. (2021). DeepOmix: A scalable and interpretable multi-omics deep learning framework and application in cancer survival analysis. Comput. Struct. Biotechnol. J..

[60] Moon S., Lee H. (2022). MOMA: A multi-task attention learning algorithm for multi-omics data interpretation and classification. Bioinformatics.

[61] Desautels T., Calvert J., Hoffman J., Jay M., Kerem Y., Shieh L., Shimabukuro D., Chettipally U., Feldman M.D., Barton C. (2016). Prediction of Sepsis in the Intensive Care Unit With Minimal Electronic Health Record Data: A Machine Learning Approach. JMIR Med. Inform..

[62] Rosnati M., Fortuin V. (2021). MGP-AttTCN: An interpretable machine learning model for the prediction of sepsis. PLoS ONE.

[63] Kim T., Tae Y., Yeo H.J., Jang J.H., Cho K., Yoo D., Lee Y., Ahn S.H., Kim Y., Lee N. (2023). Development and Validation of Deep-Learning-Based Sepsis and Septic Shock Early Prediction System (DeepSEPS) Using Real-World ICU Data. J. Clin. Med..

[64] Sadasivuni S., Saha M., Bhanushali S.P., Banerjee I., Sanyal A. Real-time sepsis prediction using fusion of on-chip analog classifier and electronic medical record. Proceedings of the 2022 IEEE International Symposium on Circuits and Systems (ISCAS).

[65] Yagin F.H., Aygun U., Algarni A., Colak C., Al-Hashem F., Ardigò L.P. (2024). Platelet Metabolites as Candidate Biomarkers in Sepsis Diagnosis and Management Using the Proposed Explainable Artificial Intelligence Approach. J. Clin. Med..

[66] Li M., Huang H., Ke C., Tan L., Wu J., Xu S., Tu X. (2022). Identification of a novel four-gene diagnostic signature for patients with sepsis by integrating weighted gene co-expression network analysis and support vector machine algorithm. Hereditas.

[67] Wang Q., Wang C., Zhang W., Tao Y., Guo J., Liu Y., Liu Z., Liu D., Mei J., Chen F. (2023). Identification of biomarkers related to sepsis diagnosis based on bioinformatics and machine learning and experimental verification. Front. Immunol..

[68] Hayashi N., Sawada Y., Ujimoto K., Yamaguchi S., Sato Y., Miki T., Nakada T., Iba T. (2021). Diagnosis of Sepsis by AI-Aided Proteomics Using 2D Electrophoresis Images of Patient Serum Incorporating Transfer Learning for Deep Neural Networks. Appl. Sci..

[69] Sweeney T.E., Azad T.D., Donato M., Haynes W.A., Perumal T.M., Henao R., Bermejo-Martin J.F., Almansa R., Tamayo E., Howrylak J.A. (2018). Unsupervised Analysis of Transcriptomics in Bacterial Sepsis Across Multiple Datasets Reveals Three Robust Clusters. Crit. Care Med..

[70] Hu C., Li Y., Wang F., Peng Z. (2022). Application of Machine Learning for Clinical Subphenotype Identification in Sepsis. Infect. Dis. Ther..

[71] Wu J., Liang J., An S., Zhang J., Xue Y., Zeng Y., Li L., Luo J. (2022). Novel biomarker panel for the diagnosis and prognosis assessment of sepsis based on machine learning. Biomark. Med..

[72] Hu C., Li L., Huang W., Wu T., Xu Q., Liu J., Hu B. (2022). Interpretable Machine Learning for Early Prediction of Prognosis in Sepsis: A Discovery and Validation Study. Infect. Dis. Ther..

[73] Kosyakovsky L.B., Somerset E., Rogers A.J., Sklar M., Mayers J.R., Toma A., Szekely Y., Soussi S., Wang B., Fan C.S. (2022). Machine learning approaches to the human metabolome in sepsis identify metabolic links with survival. Intensive Care Med. Exp..

[74] Ho C.S., Jean N., Hogan C.A., Blackmon L., Jeffrey S.S., Holodniy M., Banaei N., Saleh A.A.E., Ermon S., Dionne J. (2019). Rapid identification of pathogenic bacteria using Raman spectroscopy and deep learning. Nat. Commun..

[75] Shahn Z., Shapiro N.I., Tyler P.D., Talmor D., Lehman L.H. (2020). Fluid-limiting treatment strategies among sepsis patients in the ICU: A retrospective causal analysis. Crit. Care.

[76] Langley R.J., Tsalik E.L., van Velkinburgh J.C., Glickman S.W., Rice B.J., Wang C., Chen B., Carin L., Suarez A., Mohney R.P. (2013). An integrated clinico-metabolomic model improves prediction of death in sepsis. Sci. Transl. Med..

[77] Rivers E., Nguyen B., Havstad S., Ressler J., Muzzin A., Knoblich B., Peterson E., Tomlanovich M. (2001). Early goal-directed therapy in the treatment of severe sepsis and septic shock. N. Engl. J. Med..

[78] Seymour C.W., Gesten F., Prescott H.C., Friedrich M.E., Iwashyna T.J., Phillips G.S., Lemeshow S., Osborn T., Terry K.M., Levy M.M. (2017). Time to Treatment and Mortality during Mandated Emergency Care for Sepsis. N. Engl. J. Med..

[79] Jin X., Shen H., Zhou P., Yang J., Yang S., Ni H., Yu Y., Zhang Z. (2025). Research Progress on Sepsis Diagnosis and Monitoring Based on Omics Technologies: A Review. Diagnostics.

[80] Zheng L., Lin F., Zhu C., Liu G., Wu X., Wu Z., Zheng J., Xia H., Cai Y., Liang H. (2020). Machine Learning Algorithms Identify Pathogen-Specific Biomarkers of Clinical and Metabolomic Characteristics in Septic Patients with Bacterial Infections. BioMed Res. Int..

[81] Kollef M.H., Shorr A.F., Bassetti M., Timsit J.F., Micek S.T., Michelson A.P., Garnacho-Montero J. (2021). Timing of antibiotic therapy in the ICU. Crit. Care.

[82] Feretzakis G., Loupelis E., Sakagianni A., Kalles D., Martsoukou M., Lada M., Skarmoutsou N., Christopoulos C., Valakis K., Velentza A. (2020). Using Machine Learning Techniques to Aid Empirical Antibiotic Therapy Decisions in the Intensive Care Unit of a General Hospital in Greece. Antibiotics.

[83] Garnica O., Gómez D., Ramos V., Hidalgo J.I., Ruiz-Giardín J.M. (2021). Diagnosing hospital bacteraemia in the framework of predictive, preventive and personalised medicine using electronic health records and machine learning classifiers. EPMA J..

[84] Hyland S.L., Faltys M., Hüser M., Lyu X., Gumbsch T., Esteban C., Bock C., Horn M., Moor M., Rieck B. (2020). Early prediction of circulatory failure in the intensive care unit using machine learning. Nat. Med..

[85] Gao Z., Li Y. (2023). Enhancing single-cell biology through advanced AI-powered microfluidics. Biomicrofluidics.

[86] Papareddy P., Lobo T.J., Holub M., Bouma H., Maca J., Strodthoff N., Herwald H. (2025). Transforming sepsis management: AI-driven innovations in early detection and tailored therapies. Crit. Care.

[87] Alzubaidi L., Bai J., Al-Sabaawi A., Santamaría J., Albahri A.S., Al-dabbagh B.S.N., Fadhel M.A., Manoufali M., Zhang J., Al-Timemy A.H. (2023). A survey on deep learning tools dealing with data scarcity: Definitions, challenges, solutions, tips, and applications. J. Big Data.

[88] Levy M.M., Fink M.P., Marshall J.C., Abraham E., Angus D., Cook D., Cohen J., Opal S.M., Vincent J.L., Ramsay G. (2003). 2001 SCCM/ESICM/ACCP/ATS/SIS International Sepsis Definitions Conference. Intensive Care Med..

[89] Huang G., Laradji I., Vázquez D., Lacoste-Julien S., Rodríguez P. (2023). A Survey of Self-Supervised and Few-Shot Object Detection. IEEE Trans. Pattern Anal. Mach. Intell..

[90] Ennab M., McHeick H. (2022). Designing an Interpretability-Based Model to Explain the Artificial Intelligence Algorithms in Healthcare. Diagnostics.

[91] Bienefeld N., Boss J.M., Lüthy R., Brodbeck D., Azzati J., Blaser M., Willms J., Keller E. (2023). Solving the explainable AI conundrum by bridging clinicians’ needs and developers’ goals. NPJ Digit. Med..

[92] Toussaint P.A., Leiser F., Thiebes S., Schlesner M., Brors B., Sunyaev A. (2023). Explainable artificial intelligence for omics data: A systematic mapping study. Brief. Bioinform..

[93] Strudwick J., Gardiner L.-J., Denning-James K., Haiminen N., Evans A., Kelly J., Madgwick M., Utro F., Seabolt E., Gibson C. (2024). AutoXAI4Omics: An automated explainable AI tool for omics and tabular data. Brief. Bioinform..

[94] Cheng H., Zhang M., Shi J.Q. (2024). A Survey on Deep Neural Network Pruning: Taxonomy, Comparison, Analysis, and Recommendations. IEEE Trans. Pattern Anal. Mach. Intell..

[95] Wang L., Yoon K.J. (2022). Knowledge Distillation and Student-Teacher Learning for Visual Intelligence: A Review and New Outlooks. IEEE Trans. Pattern Anal. Mach. Intell..

[96] Price W.N., Cohen I.G. (2019). Privacy in the age of medical big data. Nat. Med..

[97] Obermeyer Z., Powers B., Vogeli C., Mullainathan S. (2019). Dissecting racial bias in an algorithm used to manage the health of populations. Science.

[98] Rajkomar A., Hardt M., Howell M.D., Corrado G., Chin M.H. (2018). Ensuring Fairness in Machine Learning to Advance Health Equity. Ann. Intern. Med..

[99] Lazar C., Meganck S., Taminau J., Steenhoff D., Coletta A., Molter C., Weiss-Solís D.Y., Duque R., Bersini H., Nowé A. (2013). Batch effect removal methods for microarray gene expression data integration: A survey. Brief. Bioinform..

[100] Leek J.T., Scharpf R.B., Bravo H.C., Simcha D., Langmead B., Johnson W.E., Geman D., Baggerly K., Irizarry R.A. (2010). Tackling the widespread and critical impact of batch effects in high-throughput data. Nat. Rev. Genet..

[101] Wang J. (2025). ComBat-met: Adjusting batch effects in DNA methylation data. NAR Genom. Bioinform..

